# Degradation of aggregated LDL occurs in complex extracellular sub-compartments of the lysosomal synapse

**DOI:** 10.1242/jcs.181743

**Published:** 2016-03-01

**Authors:** Rajesh K. Singh, Valéria C. Barbosa-Lorenzi, Frederik W. Lund, Inna Grosheva, Frederick R. Maxfield, Abigail S. Haka

**Affiliations:** Department of Biochemistry, Weill Cornell Medical College, New York, NY 10065, USA

**Keywords:** Aggregated LDL, Extracellular catabolism, F-actin, Macrophage

## Abstract

Monocyte-derived cells use an extracellular, acidic, lytic compartment (a lysosomal synapse) for initial degradation of large objects or species bound to the extracellular matrix. Akin to osteoclast degradation of bone, extracellular catabolism is used by macrophages to degrade aggregates of low density lipoprotein (LDL) similar to those encountered during atherogenesis. However, unlike osteoclast catabolism, the lysosomal synapse is a highly dynamic and intricate structure. In this study, we use high resolution three dimensional imaging to visualize compartments formed by macrophages to catabolize aggregated LDL. We show that these compartments are topologically complex, have a convoluted structure and contain sub-regions that are acidified. These sub-regions are characterized by a close apposition of the macrophage plasma membrane and aggregates of LDL that are still connected to the extracellular space. Compartment formation is dependent on local actin polymerization. However, once formed, compartments are able to maintain a pH gradient when actin is depolymerized. These observations explain how compartments are able to maintain a proton gradient while remaining outside the boundaries of the plasma membrane.

## Abstract (precis)

**Summary:** Macrophages use extracellular compartments, termed lysosomal synapses, that are topologically complex, have a convoluted structure and contain sub-regions that are acidified to digest aggregated lipoproteins.

## INTRODUCTION

Our laboratory and others have described a process in which large moieties or species tightly bound to the extracellular matrix are initially digested in an extracellular, acidic, lytic compartment (Zhang et al., 1997; Grosheva et al., 2009; Haka et al., 2009). We describe this process as exophagy. We have studied exophagy in the context of macrophage degradation of aggregated low density lipoproteins (agLDL) as occurs during atherogenesis (Grosheva et al., 2009; Haka et al., 2009). The macrophages create deeply invaginated, actively acidified structures in which extracellular agLDL is digested by exocytosed lysosomal enzymes (lysosomal synapses) (Haka et al., 2009). Exophagic catabolism of agLDL results in uptake of cholesterol by the macrophage, leading to foam cell formation, a hallmark of early atherogenesis.

This degradative mechanism, used by macrophages to allow lysosomal hydrolases to digest large objects, somewhat parallels the mechanism used by osteoclasts to degrade bone (Baron et al., 1985; Stenbeck, 2002; Jurdic et al., 2006). However, there are several notable distinctions between osteoclast catabolism of bone and macrophage catabolism of agLDL. Notably, bone is a rigid surface that cannot be deformed easily. Therefore, osteoclasts make extensive membrane invaginations (the ruffled border) to maximize the degradative surface area into which they can secrete lysosomal enzymes. By contrast, agLDL can be deformed more easily, allowing the macrophage to manipulate and sequester sub-portions of the aggregate in surface-connected compartments prior to degradation. Differences between osteoclasts and macrophages are evident in the properties of the extracellular compartments they create. Osteoclasts form a stable compartment surrounded by an actin ring (Chellaiah, 2005). Tight binding of osteoclast integrins, particularly α_v_β_3_, to RGD-containing proteins in the bone surface inhibits the diffusion of protons, thereby facilitating the generation of a stable acidic environment necessary for the activity of exocytosed lysosomal hydrolases (Nakamura et al., 1999; McHugh et al., 2000; Luxenburg et al., 2007). We have observed a low pH in the extracellular compartments formed by macrophages, but the pH fluctuates over time (Haka et al., 2009). This suggests that these compartments are dynamic and likely open and close. This would allow catabolic products such as free cholesterol to be transferred into the macrophage or released extracellularly. Cholesterol transfer into the macrophage plasma membrane stimulates many acute events, including actin polymerization and macropinocytosis, which likely promotes uptake of partially catabolized LDL into the cell and subsequent foam cell formation (Kruth et al., 2005; Qin et al., 2006; Nagao et al., 2007; Grosheva et al., 2009). Release of free cholesterol from the agLDL-containing compartment has been demonstrated biochemically (Haka et al., 2009). In advanced atherosclerotic lesions, deposition of extracellular cholesterol crystals in vessel walls is known to occur, but the origin of this cholesterol is unknown. Macrophage catabolism of agLDL and subsequent release of free cholesterol extracellularly might provide a source of free cholesterol for the formation of such crystals.

The biology described herein is also somewhat akin to frustrated phagocytosis, a pro-inflammatory type of macrophage activation characterized by extracellular release of lysosomal contents and cytoskeletal actin rearrangements when the macrophage is facing a target much larger than itself (Labrousse et al., 2011; Singh et al., 2014). Large crystals, such as monosodium urate, or crystalline fibers, such as asbestos, remain trapped at the cell surface as the macrophage attempts their clearance. In the setting of atherosclerosis, the ability of macrophages to catabolize and ingest agLDL is not ‘frustrated’ and eventually results in foam cell formation. Thus, the use of an extracellular, acidic, lytic compartment for degradation might be either pro-inflammatory (e.g. airborne pollutants, gout, atherosclerosis) or non-inflammatory (e.g. normal bone remodeling). There are likely to be many more examples of this type of process in a variety of biological contexts, thus highlighting the need for a detailed understanding of the morphology and function of extracellular catabolic compartments.

Although much has been learned about the compartment that macrophages use to catabolize agLDL, many questions remain unanswered. As summarized above, we have found that macrophage catabolism of agLDL occurs extracellularly. If this is the case, how are compartments able to acidify? It is reasonable to suggest that protons would simply diffuse into the extracellular space. Are compartments really extracellular or are acidified portions of the compartment fully sealed from the extracellular space? Further, although it is known that actin polymerization is required for the formation of the compartment, what is its role in this process? To answer these questions, in this study we investigate the morphology and formation of compartments formed for exophagy using high resolution microscopy techniques.

Using electron and high resolution optical microscopy, we now show that compartments used for exophagic degradation (lysosomal synapses) conform to the aggregate and contain sub-regions that are acidified. These sub-regions are characterized by a close apposition of the macrophage plasma membrane and agLDL, while still retaining connectivity to extracellular portions of the aggregate. Sub-compartments are able to exclude large molecules, such as cholera toxin B (CtB), indicating that they experience limited diffusion to the extracellular space. Compartment formation is dependent on local actin polymerization. However, once formed, compartments are able to maintain a pH gradient even when actin is depolymerized, indicating that receptor binding likely mediates tight sealing between the macrophage plasma membrane and aggregate. These observations highlight a role for actin in the formation of the compartments and explain how the lysosomal synapse is able to maintain a proton gradient while remaining outside the boundaries of the plasma membrane. To our knowledge, this is a unique and newly identified example of a convoluted, partially acidified, catabolic structure that is sequestered but also extracellular.

## RESULTS

### AgLDL is sequestered in acidic compartments that are surface-connected

**Fig. 1.**
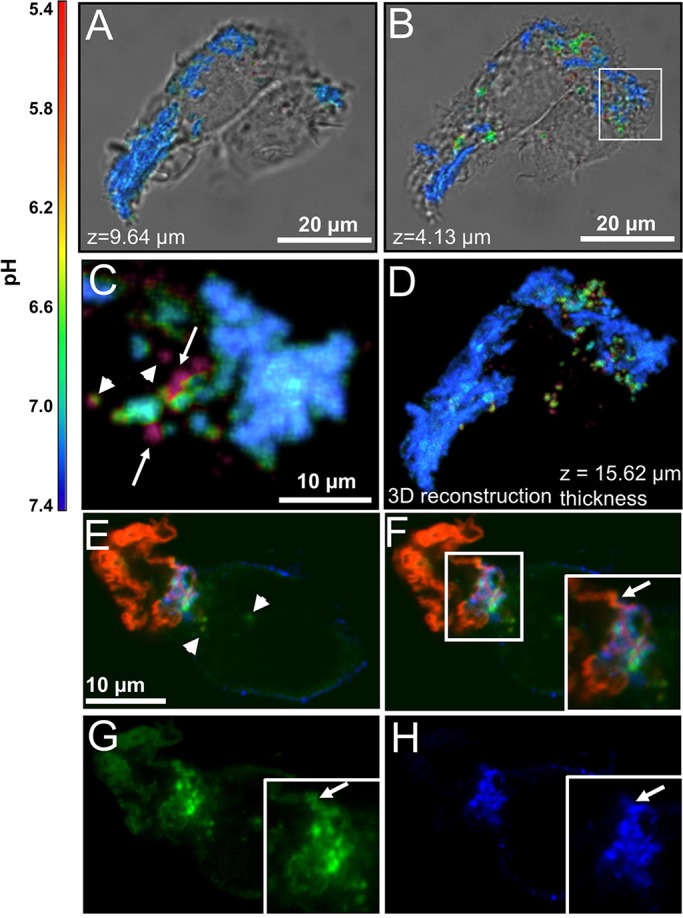
**AgLDL is sequestered in compartments that are surface-connected and contain acidified sub-regions where free cholesterol is generated.** (A–D) Ratiometric confocal images of J774 cells incubated with agLDL dual-labeled with a pH-sensitive and pH-insensitive probe for 1 h. (A) Aggregate contained above the surface of the cells and (B) agLDL in the middle of the cells. (C) Enlargement of an area containing acidified regions, highlighted by a box in B. Regions of acidification that represent completely internalized vesicles (arrowheads) and areas of low pH that retain their connection to portions of the aggregate that are at neutral pH (arrows) can be seen. (D) 3D reconstruction of the data shown in A and B. (E–H) RAW264.7 macrophage-like cells were incubated with Alexa633–agLDL (red) for 1 h, stained with Alexa555–CtB (blue) on ice to label the plasma membrane, fixed and stained with filipin (green) to visualize free cholesterol. (E) A cell is contacting Alexa633–agLDL. Some filipin labeling of free cholesterol can be seen inside the cell (arrowheads). (F–H) An extracellular strand of agLDL in close proximity to the cells is shown by an arrow in the inset (F). Single color images of the same region show that it is labeled with filipin (G), but the cell only touches this strand at the base (H). There are areas of filipin labeling that are also labeled with CtB, suggesting that these are also associated with the plasma membrane.

We have previously shown that when macrophages interact with agLDL, regions of low pH are observed at contact sites (Haka et al., 2009). Compartment acidification depends on the activity of plasma membrane vacuolar (H^+^)-ATPases (Haka et al., 2009), which transport protons from the cytoplasm to the extracellular space. Although we hypothesized that these acidified zones occurred in sub-regions of the compartment that were almost sealed but still extracellular, it was difficult to understand how the cell could maintain an extracellular proton gradient. To further investigate these low pH regions, J774a.1 macrophage-like cells (hereafter referred to as J774 cells) were incubated for 1 h with agLDL labeled with a pH-sensitive and a pH-insensitive fluorophore. The pH at points of contact between the aggregate and macrophage was determined by ratiometric live cell imaging. Fig. 1A shows a single *x*,*y*-slice from a confocal stack at a position above the surface of the cells, and no acidification of this portion of the agLDL is observed. By contrast, Fig. 1B shows a *x*,*y*-slice taken through the cell. At this depth, several acidic regions surrounding the aggregate can be seen. Fig. 1C displays an enlargement of an area containing acidified regions from the *x*,*y*-slice taken through the cell. Although some regions of acidification represent completely internalized vesicles (arrowheads, Fig. 1C), other areas of low pH still retain their connection to portions of the aggregate that are at neutral pH and remain extracellular (arrows, Fig. 1C). A three-dimensional (3D) reconstruction of these data (Fig. 1D; Movie 1) shows that small sub-regions of the agLDL are exposed to low pH, and many of these regions retain connectivity to extracellular portions of the aggregate.

### Cholesteryl ester hydrolysis occurs in focal regions of the lysosomal synapse

The lysosomal synapse functions as an extracellular degradative organelle in which free cholesterol is generated by the action of lysosomal acid lipase (LAL) on cholesteryl esters contained in the aggregate (Haka et al., 2009). In order for LAL to function optimally, an acidic environment is required (Ameis et al., 1994). To examine the generation of free cholesterol in the lysosomal synapse, we used filipin, a fluorescent sterol-binding polyene that can be used for detection of free cholesterol (Qin et al., 2006). RAW264.7 macrophage-like cells were incubated with Alexa-Fluor-633-conjugated agLDL (Alexa633–agLDL), stained with Alexa555–CtB on ice to label the plasma membrane, and fixed with 3% paraformaldehyde (PFA). When cells are fixed with PFA, substantial mobility of lipid-linked proteins is maintained (Mayor et al., 1994). This allows visualization of the topological organization of the lysosomal synapse as the labeled CtB diffuses into the contact zone between the macrophage and the aggregate. Using this approach, we observe filipin staining (green, Fig. 1E–G, arrow in F,G) in the region of contact with the agLDL (red) that is in contact with the plasma membrane (blue, Fig. 1E,F,H). The presence of plasma membrane around areas of intense filipin staining indicates that cholesteryl ester hydrolysis is occurring in an extracellular compartment as reported previously (Haka et al., 2009). The generation of free cholesterol in the lysosomal synapse is focal and appears to occur in sub-regions of the compartment, likely those same areas that are acidified.

### The lysosomal synapse contains sub-compartments with narrow openings to the extracellular space

The ratiometric live cell imaging indicates that agLDL is contained in acidic compartments that retain surface connectivity. However, to maintain a low pH, the macrophage would have to sequester portions of the aggregate in sub-compartments able to limit diffusion of protons into the extracellular space. To examine the architecture of compartments formed during macrophage catabolism of agLDL in greater detail, we used electron microscopy of serial sections and focused-ion beam scanning electron microscopy (FIB-SEM).

**Fig. 2.**
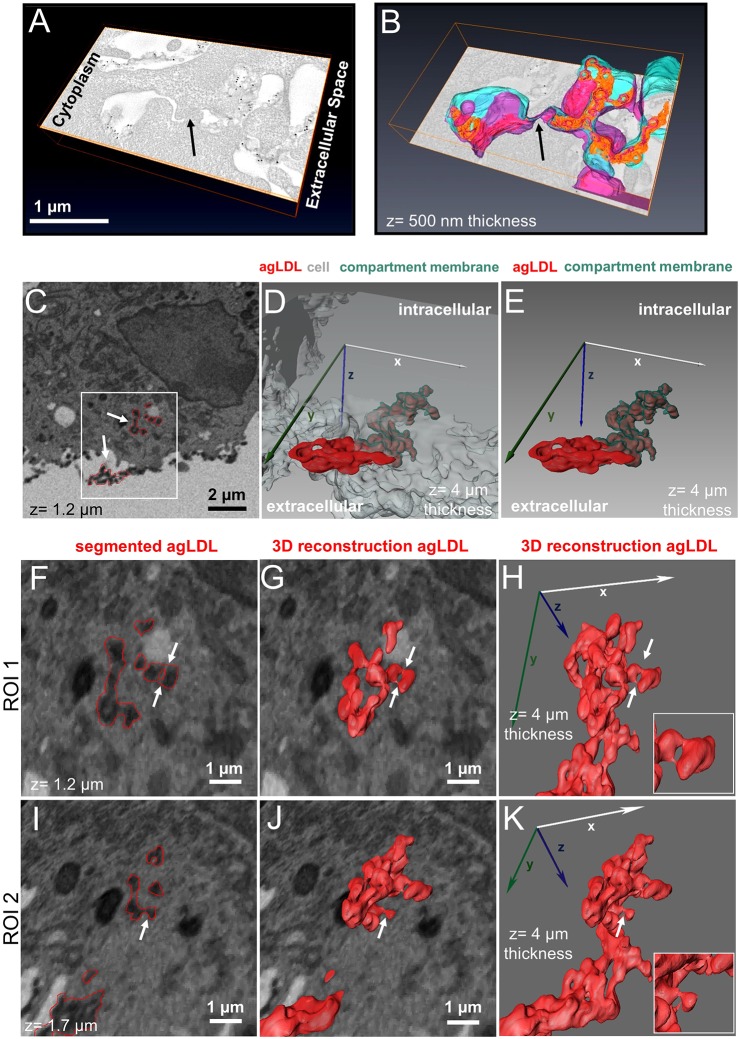
**Electron microscopy reveals agLDL in surface-connected compartments containing sub-regions with narrow openings to the extracellular space.** (A–B) J774 cells were incubated with colloidal-gold-labeled agLDL for 1 h, prepared for electron microscopy, 250-nm sections cut and images were acquired at different tilt angles. (A) A detailed view of a macrophage lysosomal synapse in a single computed electron microscopy slice. (B) A 3D reconstruction of the macrophage plasma membrane and agLDL generated from two serial sections. Blue shows the outer leaflet of the plasma membrane, purple denotes the inner leaflet, orange represents agLDL contained in the compartment and pink shows agLDL visualized through the plasma membrane. The compartment is continuous with the extracellular space but a close apposition of the macrophage membrane (arrows in A,B) was observed. (C–K) FIB-SEM data acquired from a J774 cell interacting with colloidal-gold-labeled agLDL for 1 h prior to fixation and embedding. Segmentation of the aggregate was carried out in one hundred slices (total *z* size 4 µm). (C) An SEM image of a macrophage interacting with agLDL. Arrows show colloidal-gold-labeled agLDL and regions of aggregate segmentation are overlaid in red. (D) A 3D reconstruction of the segmented agLDL shows that it is continuous with the extracellular space and (E) that sequestered portions of agLDL are contained in an invaginated compartment tightly surrounded by plasma membrane. (F–K) Enlargements of a region, highlighted by a box in C [upper arrow in box indicates ROI 1 at 1.2 μm depth (F–H), ROI 2 indicates the same region at 1.7 µm depth (I–K); the lower arrow highlights another region containing a portion of the aggregate], reveal smaller areas within the main compartment that are pinched and narrowed but preserve connectivity to the extracellular space (arrows, insets). ROI, region of interest.

J774 cells were incubated with colloidal-gold-labeled agLDL, fixed and prepared for electron microscopy. We performed electron microscopy tomography (Winkler and Taylor, 2006) on 250-nm sections. Fig. 2A shows a detailed view of a macrophage forming a lysosomal synapse in response to agLDL in a single computed electron microscopy slice. A preliminary example of this electron microscopy tomography analysis was described previously (Haka et al., 2009). A very complex topographical surface, including invaginations containing portions of agLDL, is observed in the region of contact between the macrophage and aggregate. Using data from tomographic analysis of serial sections (Movie 2), a 3D reconstruction of the macrophage plasma membrane and aggregate was generated (Fig. 2B). In this reconstruction, blue represents the outside of the macrophage plasma membrane and purple shows the cytoplasmic leaflet. Aggregate appears in orange, unless visualized through the membrane, in which case it is pink. The compartment is clearly continuous with the extracellular space, but a close apposition of the macrophage membrane (arrows, Fig. 2A,B) was observed. An image of the entire cell and the area used for 3D reconstruction are also shown (Fig. S1A,B). Although the data set did not include sufficient serial sections to enable reconstruction of the entire compartment in 3D, almost-sealed regions like this might explain how the lysosomal synapse is able to maintain a proton gradient while remaining outside the boundaries of the plasma membrane.

Understanding the properties of the lysosomal synapse requires a full high-resolution 3D image of these compartments. Because of their size, capturing a compartment in its entirety with electron microscopy tomography of serial sections is challenging (each section is only 250-nm thick). In order to examine a complete lysosomal synapse with high resolution in 3D, we employed FIB-SEM. FIB-SEM images were acquired from J774 cells incubated with colloidal-gold-labeled agLDL for 1 h, and we confirmed that these images resembled those generated by TEM (Fig. S1C,D). We generated a stack of 990 FIB-SEM images with 40 nm separating each image, and we identified agLDL contained within the lysosomal synapse that was seen to be connected with the extracellular space, as shown in Fig. 2C–K. Fig. 2C shows an image of a macrophage interacting with agLDL. Regions of agLDL are outlined in red. A 3D representation of the lysosomal synapse highlighted by a box in Fig. 2C was generated using image processing software to display agLDL and the cellular plasma membrane forming the compartment. Fig. 2D,E shows both extracellular agLDL and the portion contained in deep membrane invaginations (Movie 3). Plasma membrane tightly surrounds the agLDL contained within the lysosomal synapse (Fig. 2E). Close examination of the compartment reveals smaller areas within the main compartment that are pinched and narrowed but preserve connectivity to the extracellular space (Fig. 2F–K). These sequestered areas morphologically resemble the regions of low pH and free cholesterol generation seen in Fig. 1 and likely experience very limited diffusion to the extracellular space. Taken together these data indicate that topologically extracellular agLDL is sequestered in severely restricted narrow areas deep in the cell that would likely be capable of maintaining a low pH.

### AgLDL resides in sub-compartments that retain surface connectivity but have restricted permeability to the extracellular space

**Fig. 3.**
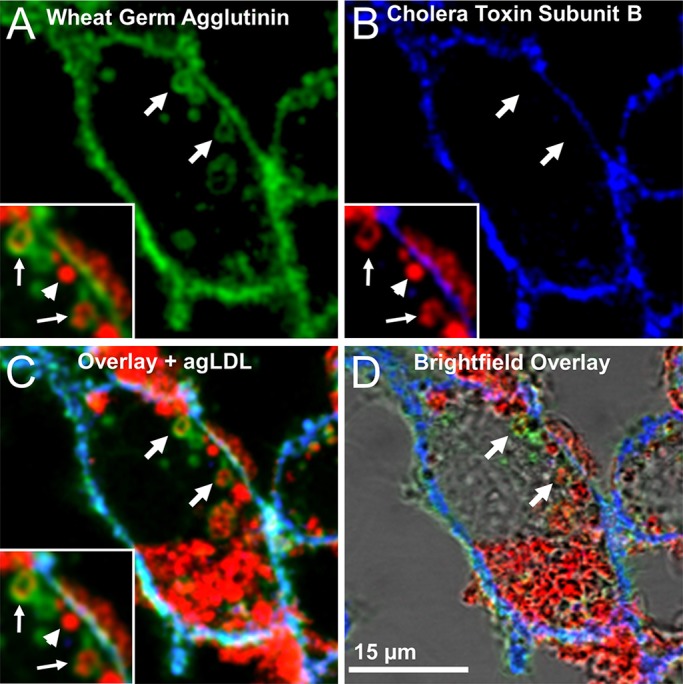
**AgLDL resides in sub-compartments that retain surface connectivity but have restricted access to the extracellular space.** J774 cells were incubated with Alexa633–agLDL for 1 h, stained with Alexa555–CtB on ice for 5 min, fixed with 3% PFA and 0.5% glutaraldehyde to block the diffusion of lipid-linked proteins (CtB) following fixation and then labeled with Alexa488–WGA. (A) WGA staining shows compartments (arrows) that contain agLDL (red, inset) and are surface connected. Some regions of agLDL represent completely internalized vesicles that are negative for WGA (arrowhead, inset). (B) CtB staining labels the surface of the cell but was largely excluded from areas of agLDL sequestration (blue, arrows, inset). (C) Overlay and (D) brightfield overlay of WGA staining, CtB staining and agLDL, showing that agLDL resides in surface-connected compartments that seal sufficiently to exclude CtB (C, arrows, inset).

Having observed narrowed regions of the lysosomal synapse by FIB-SEM, we examined how permeable these areas are to the extracellular space. To more directly assess this, J774 cells were incubated with Alexa633–agLDL for 1 h. Live cells were then treated with Alexa555–CtB on ice, washed, fixed with 3% PFA and 0.5% glutaraldehyde and finally labeled with Alexa488–wheat-germ-agglutinin (WGA). In this experiment, both the WGA and CtB are used to label the macrophage plasma membrane. When the fixative includes 0.5% glutaraldehyde in addition to PFA, diffusion of lipid-linked proteins following fixation is inhibited (Mayor et al., 1994; Kusumi and Suzuki, 2005), and the lipid-bound CtB would be unable to redistribute to the lysosomal synapse after fixation. Any CtB staining in the lysosomal synapse will therefore represent direct access of CtB to the lysosomal synapse. The WGA is introduced following fixation, which results in mild permeabilization, so WGA is able to access plasma membrane even in tightly sealed sub-compartments. Fig. 3A shows the plasma membrane labeled by WGA. Although some regions of agLDL (red) represent completely internalized vesicles (arrowhead, inset, Fig. 3A), other areas of aggregate are surrounded by plasma membrane (green) and contained in the lysosomal synapse (arrows, Fig. 3A). Alexa555–CtB staining was seen at the surface of the macrophage, but was largely excluded from areas of agLDL sequestration (arrows, inset, Fig. 3B). Sub-compartments that exhibit WGA staining but are negative for CtB (arrows, inset, Fig. 3C,D) demonstrate that agLDL resides in surface-connected compartments that seal sufficiently to exclude molecules of 57 kDa in size and larger. We note that fixation might alter the permeability of the lysosomal synapse in this experiment. However, the CtB was added before fixation, so effects of fixation would not alter the exclusion that we observe in glutaraldehyde-fixed cells. Unfortunately, because the compartments are dynamic (Haka et al., 2009), we cannot carry out longer term experiments to characterize the permeability barrier in detail. The ability to maintain a pH gradient suggests that there is at least a transient barrier to diffusion of very small molecules.

### Macrophages make intricate F-actin structures that surround and constrict portions of agLDL

**Fig. 4.**
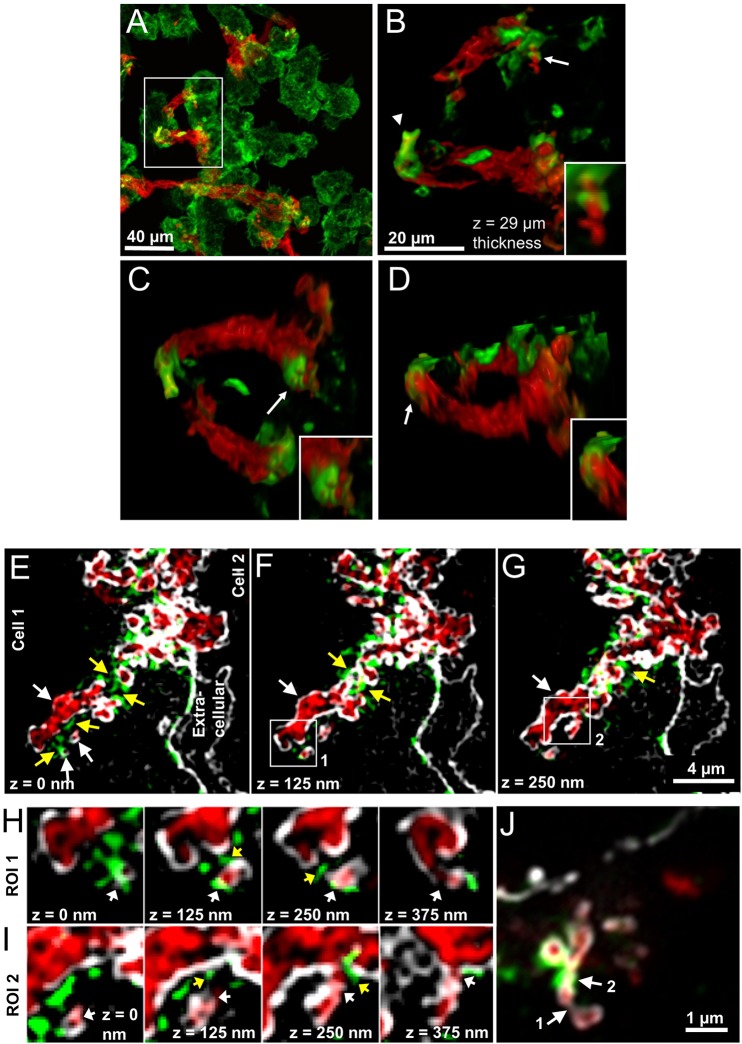
**Macrophages make intricate F-actin structures that promotes macrophage plasma membrane contact with agLDL.** (A–D) BMMs were incubated with Alexa546–agLDL for 1 h, fixed and stained with Alexa488–phalloidin to visualize F-actin. (A) A maximum projection image showing macrophages forming F-actin-rich structures (green) in areas of contact with agLDL (red). (B–D) Still images from a 3D reconstruction of the area highlighted by a box in A. (B) An F-actin cap surrounds a portion of agLDL (arrowhead) and a ring of F-actin constricts the aggregate (arrow, inset). (C) Adjacent ring-like F-actin structures surrounding portions of agLDL (arrow, inset). (D) A 180° rotation of the image shown in B displays finger-like F-actin structures in areas of contact with agLDL (arrow, inset). (E–J) J774 cells were incubated with Alexa405–agLDL for 1 h, labeled with Alexa488–CtB on ice, fixed with 3% PFA, labeled with Alexa555–phalloidin to stain F-actin and 3D-SIM images acquired. (E) AgLDL (red) is contained between two cells but is deeply invaginated into cell 1. The presence of plasma membrane (white) surrounding agLDL confirms that the aggregate is contained in an extracellular compartment. Three agLDL-containing sub-compartments (white arrows) are sequestered from the main lysosomal synapse by F-actin-rich structures (yellow arrows). Two ROIs, highlighted by boxes in F (ROI 1) and G (ROI 2), are show in detail in H and I, respectively. Arrows as for E–G. (J) Airyscan-confocal microscopy image of a lysosomal synapse showing examples of narrow junctions in the compartment. Some pinched and narrowed parts of the synapse appear to lack actin (arrow 1) whereas in others, F-actin is associated with these areas (arrow 2).

We have previously shown that compartments used for exophagy are formed by F-actin-driven membrane protrusions surrounding the aggregate (Grosheva et al., 2009). However, we had not previously investigated the relationship between these F-actin structures and the structure of the lysosomal synapse. To do this, bone marrow-derived macrophages (BMMs) were incubated with Alexa546–agLDL prior to fixation and staining with Alexa488–phalloidin to visualize F-actin. Fig. 4A displays a maximum intensity projection image of a confocal *z*-stack, showing several macrophages interacting with agLDL (red). Fig. 4B–D shows images from a 3D reconstruction (Movie 4) of the area highlighted by a box in Fig. 4A. The 3D reconstruction illustrates examples of elaborate F-actin structures (green) that are made by three macrophages interacting with agLDL. An F-actin cap that completely surrounds a portion of agLDL (arrowhead, Fig. 4B) and a ring of F-actin that constricts the aggregate (arrow and inset, Fig. 4B) are visualized in the 3D reconstruction. These actin structures appear to pinch and sequester the agLDL into more sealed regions and to bring the macrophage plasma membrane into contact with the aggregate. The arrow and inset in Fig. 4C show adjacent rings of F-actin that surround the aggregate. Fig. 4D is a 180° rotation of the image shown in Fig. 4B and shows ‘fingers’ of F-actin (arrow and inset), perhaps used by the macrophage to promote membrane interaction with the agLDL. These data show that F-actin promotes cell contact with the aggregate and aids the formation of sub-compartments within the lysosomal synapse.

### Actin polymerization promotes macrophage plasma membrane contact with agLDL and sequestration of the aggregate

We further investigated the relationship between F-actin structures and plasma membrane contact with agLDL in the lysosomal synapse. To examine the macrophage plasma membrane in regions of actin polymerization and compartment narrowing, we incubated J774 cells with Alexa405–agLDL and labeled them with Alexa555–CtB on ice prior to fixation with 3% PFA, to allow redistribution of CtB into the compartment. Cells were then incubated with Alexa488–phalloidin to stain F-actin and imaged using two high resolution microscopy techniques, 3D-structured illumination microscopy (SIM) or Airyscan confocal microscopy. Fig. 4E–G shows 3D-SIM images of a lysosomal synapse. The aggregate (red) is contained between two cells but is deeply invaginated into cell 1. The macrophage plasma membrane (white) shows the complex surface topography of the lysosomal synapse. The presence of plasma membrane surrounding agLDL confirms that the aggregate is contained in a compartment that is topologically outside of the cell. Three agLDL-containing sub-compartments (white arrows, Fig. 4E) are sequestered from the main lysosomal synapse by F-actin-rich structures (labeled in green, highlighted by yellow arrows, Fig. 4E–G) but are surrounded by plasma membrane, confirming that they are extracellular. These images show that actin is polymerized in regions where the compartment narrows. Further evidence of this can be seen in Movie 5, arrows 3 and 3′. Fig. 4H,I show consecutive *x*,*y*-slices of the two areas highlighted by boxes in Fig. 4F,G. White arrows show sub-compartments that are tightly surrounded by plasma membrane with very narrow, partially occluded openings to the extracellular space. Narrowing of the lysosomal synapse is often accompanied by surrounding F-actin (yellow arrows, Fig. 4F,G). These sub-compartments can also be visualized in Movie 5, arrows 1 and 2.

Fig. 4J shows a single plane from an Airyscan confocal microscopy *z*-stack. This image demonstrates the formation of very restricted junctions in the lysosomal synapse (arrows 1 and 2). Again, actin polymerization is present in areas where the membrane is pinched and in tight apposition with the aggregate, indicated by arrow 2. To visualize the structure of actin with respect to agLDL and the plasma membrane, we created a 3D surface reconstruction of the data shown in Fig. 4J (Movie 6), spanning 5.44 µm in the *z*-direction. The movie begins by showing *x*,*y* images of the plasma membrane (gray). Next, the plasma membrane and aggregate (red) are displayed in 3D. Addition of F-actin (green) to the 3D reconstruction shows that it wraps around the agLDL and plasma membrane forming the lysosomal synapse. Removal of the plasma membrane and aggregate reveals ring-like structures of F-actin, similar to those shown in Fig. 3, constricting portions of the agLDL, highlighted by circles in Movie 6. Taken together the results presented in Fig. 4 show that actin polymerization helps bring the macrophage plasma membrane into close contact with agLDL and form sequestered sub-compartments needed to support an acidic pH.

### Actin polymerization is required for compartment formation and plasma membrane contact with agLDL

**Fig. 5.**
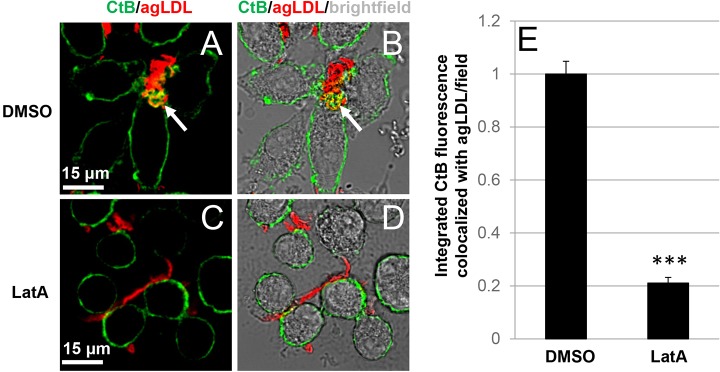
**Actin polymerization is required to form the lysosomal synapse.** (A–D) J774 cells were incubated with Alexa546–agLDL for 1 h in the presence of DMSO (A,B) or 1 μM LatA (C,D), labeled with Alexa488–CtB on ice and fixed with 3% PFA to allow compartment visualization. Membrane invaginations could be seen at sites of contact between control DMSO treated macrophages and agLDL (arrow, A,B), indicative of compartment formation. Cells treated with LatA did not form membrane invaginations at sites of contact with agLDL (C,D). (E) Quantification of total integrated CtB fluorescence colocalized with agLDL in DMSO- and LatA-treated macrophages. Data compiled from three independent experiments (*n*=53 fields per condition). ***P≤0.001 Student's *t*-test. Error bars represent the standard error of the mean.

Having shown that actin polymerization can help bring macrophage plasma membrane into contact with agLDL, we examined whether polymerization of actin is required during compartment formation. We used Latrunculin A (LatA) to depolymerize F-actin. J774 cells were incubated with Alexa546–agLDL for 1 h in the presence of DMSO or 1 μM LatA, labeled with Alexa488–CtB on ice and fixed with 3% PFA to allow redistribution of CtB into the compartment. Membrane invaginations could be seen at sites of contact between DMSO-treated macrophages and agLDL (arrow, Fig. 5A,B), demonstrating that the aggregate is contained in a compartment that is connected to the cell surface. Cells treated with LatA did not form membrane invaginations at sites of contact with agLDL, indicating that compartment formation was inhibited (Fig. 5C,D). Quantification of CtB fluorescence (plasma membrane) associated with agLDL revealed that LatA treatment resulted in an 80% reduction in the amount of plasma membrane colocalized with the aggregate (Fig. 5E). We confirmed that actin polymerization under these conditions was inhibited. After 1 h agLDL treatment in the presence of DMSO, J774 cells had robust F-actin structures (Fig. S2A,B). When 1 μM LatA was added with agLDL for 1 h, F-actin structures were completely abolished (Fig. S2C,D). Quantification of F-actin colocalized with agLDL revealed that under these conditions, F-actin was reduced to 1% of DMSO-treated cells (Fig. S2E). These data suggest that actin polymerization plays an important role in compartment formation and promotes macrophage plasma membrane contact with agLDL.

### Disruption of F-actin does not dissipate the pH gradient at the lysosomal synapse

**Fig. 6.**
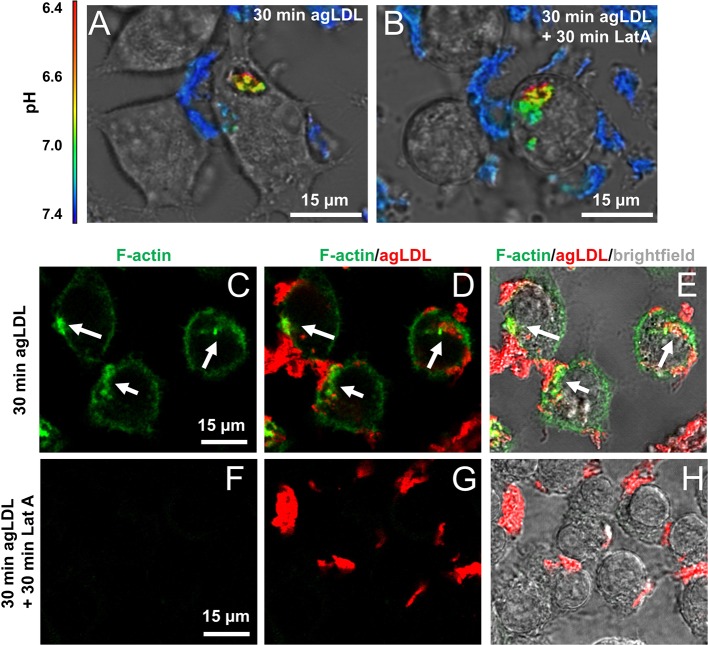
**Sustained actin polymerization is not required to maintain a pH gradient in the lysosomal synapse.** (A) Ratiometric confocal images of J774 cells interacting with agLDL dual labeled with a pH-sensitive and a pH-insensitive fluorophore for 30 min. (B) Addition of 1 μM LatA for 30 min did not abolish the low pH regions. (C–H) J774 cells were treated with agLDL for 30 min (C–E) or 30 min followed by 30 min incubation with 1 μM LatA (F–H) followed by fixation in 3% PFA and staining F-actin with Alexa488–phalloidin. Using the same treatment conditions as in B, F-actin structures at the lysosomal synapse (arrows in C−E) were completely abolished.

Although we frequently observed F-actin-rich structures in narrowed areas of the lysosomal synapse, we sometimes found pinched portions of the lysosomal synapse that were not colocalized with F-actin, which might represent areas where F-actin has disassembled (arrow 1, Fig. 4J). To see if sustained actin polymerization is necessary to maintain an established pH gradient, we measured the pH of agLDL before and after actin depolymerization. Fig. 6A shows a macrophage generating a low pH in a sub-region of a lysosomal synapse. Addition of 1 μM LatA did not abolish the low pH regions even after 30 min of treatment (Fig. 6B). Changes in cell morphology, such as rounding, are evident following addition of LatA. Further, we confirmed that acidified compartments present after LatA treatment still retain connectivity to the extracellular space (Fig. S3). Cells making acidified compartments after 30 min (Fig. S3A), were treated with LatA for 30 min (Fig. S3B), and labeled with Alexa555–CtB on ice prior to fixation with 3% PFA. CtB redistribution after fixation to the LatA-treated lysosomal synapse (Fig. S3C–E) indicated that the lysosomal synapse is still connected to the extracellular space and not fully internalized. Actin depolymerization under these conditions was also confirmed by phalloidin staining. After 30 min treatment with agLDL, cells formed robust F-actin structures at the lysosomal synapse (arrows, Fig. 6C–E). When J774 cells were incubated with agLDL for 30 min, followed by LatA for 30 min (as in Fig. 6B), F-actin structures were completely abolished (Fig. 6F–H). These data demonstrate that although actin polymerization is required for initial compartment formation, once a pH gradient is generated, F-actin is not necessary to maintain compartment sealing and a low pH environment. This suggests that F-actin facilitates close contact between the macrophage plasma membrane and aggregate to allow receptor-mediated binding which would subsequently maintain a nearly sealed compartment.

Previous data have suggested a role for low density lipoprotein receptor-related protein 1 (LRP1) in agLDL degradation (Sakr et al., 2001). Thus, we investigated a potential role for LRP1 in actin polymerization in response to agLDL. When BMMs were treated with LRP1 antagonist receptor associated protein (RAP) and then with agLDL in the presence of RAP, actin polymerization at the lysosomal synapse remained unperturbed (Fig. S4A–E). This suggests that LRP1 plays a limited role in the acute stages of macrophage response to agLDL.

## DISCUSSION

It is well known that osteoclasts create an acidic extracellular compartment that contains lysosomal hydrolases to digest bone. This is considered to be a specialized function of these cells. We have recently shown that in addition to osteoclasts, other monocyte-derived cells, such as macrophages and monocyte-derived dendritic cells, form an extracellular, acidic, lytic compartment to digest objects that cannot be internalized by standard endocytic mechanisms (Grosheva et al., 2009; Haka et al., 2009, 2015). There have also been reports that extracellular matrix can be degraded by macrophages outside of the boundary of the cell (Punturieri et al., 2000). There are likely to be many more examples of this type of process in a variety of biological settings, thus highlighting the importance of understanding these compartments.

In this study, we use high resolution imaging to visualize extracellular degradative compartments formed by different kinds of macrophages to catabolize agLDL. We use a variety of cell lines to show that exophagy is a general biological process used by macrophages and is not specific to an individual macrophage cell line. Extending the previous general characterization of lysosomal synapse biological function, we have reached a new level of understanding of its complexity and gained insight into some of its features by employing new tools. We show that compartments contain sub-regions that are acidified. These sub-regions are characterized by a close apposition of the macrophage plasma membrane and aggregate and exhibit restricted permeability to the extracellular space. The narrowed sub-compartments are surrounded by F-actin, and compartment formation is dependent on local actin polymerization. However, once formed, compartments are able to maintain a pH gradient when actin is depolymerized. This finding was somewhat surprising, given that F-actin was often associated with narrowed areas of the compartment, though we did observe pinched areas lacking F-actin staining. This suggests that F-actin might promote plasma membrane contact with agLDL that initiates macrophage receptor binding to the aggregate.

Several receptors are known to bind LDL, and the best characterized of these are scavenger receptors including CD36 and SR-A. However, conflicting evidence has emerged about their role in atherosclerosis in various mouse models, including studies indicating that CD36 and SR-A are dispensable for foam cell formation (Kunjathoor et al., 2002; Moore et al., 2005; Kuchibhotla et al., 2008; Manning-Tobin et al., 2009). *In vitro* evidence has also indicated a dispensable role for CD36, SR-A and LDL-R in agLDL degradation by macrophages (Sakr et al., 2001). The same study found a role for LRP1 in agLDL degradation (Sakr et al., 2001), suggesting macrophage binding to agLDL might occur through LRP1. However, in the current study, inhibition of LRP1 did not impair actin polymerization in response to agLDL, suggesting that another receptor might promote macrophage binding to agLDL. Another possibility is the involvement of integrins. As mentioned previously, osteoclasts use the integrin α_v_β_3_ to bind to RGD-containing proteins in bone (Nakamura et al., 1999; McHugh et al., 2000). Future studies will attempt to identify the macrophage receptors involved in binding to agLDL responsible for maintenance of a low pH in the lysosomal synapse.

Cholesterol transfer into the macrophage plasma membrane stimulates many acute events. These include activation of Rac GTPase and inactivation of RhoA, and actin-dependent formation of the lysosomal synapse is known to be dependent on Rac and/or Cdc42 but not Rho GTPase (Nagao et al., 2007; Grosheva et al., 2009). The specific proteins and their relative contributions to actin polymerization at the lysosomal synapse remain to be determined. In other processes distinct from lysosomal synapse formation but also stimulated by actin polymerization such as macrophage phagocytosis; Rac2, Cdc42 and RhoG promote FcγR-mediated phagocytosis, whereas RhoA and RhoG promote CR3-mediated phagocytosis (Caron and Hall, 1998; Tzircotis et al., 2011). One interesting difference between phagocytosis and the lysosomal synapse is that the degradative compartment between the macrophage and agLDL does not fully close. One possible explanation for this is that moieties generated during the degradation of agLDL might inhibit compartment closure. Free cholesterol in particular can activate Rac in macrophages (Swanson and Hoppe, 2004; Qin et al., 2006; Nakaya et al., 2008). Transient activation of Rac occurs during phagosome closure, and expression of constitutively active Rac results in delayed phagosome closure (Nakaya et al., 2008). Therefore, generation of free cholesterol and activation of Rac at the lysosomal synapse might delay and/or halt closure of the compartment.

A recent study characterized macrophage phagocytosis of filamentous *Legionella pneumophila* (Prashar et al., 2013)*.* The macrophage forms a long tubular phagocytic cup to which early and late endosomes and lysosomes fuse, and the macrophage uses F-actin ‘jackets’ (similar to Fig. 4B,J in this study) to limit diffusion to the outside space. Despite this, 3 kDa dextran can still diffuse from the phagocytic cup to the extracellular space, and hydrolysis does not occur in the extended phagocytic cup. The lysosomal synapse characterized in our study might therefore be akin to this modified phagocytic process specialized for long tubular species but with tighter sealing. AgLDL is likely to be more deformable than *L. pneumophila*, and this might allow closer apposition of the macrophage plasma membrane with the target. Further, we observed a series of constrictions promoted by F-actin structures and small pinched regions of the compartment (Fig. 4) that likely lead to highly restricted diffusion out of the compartment. These differences might explain why the lysosomal synapse is more tightly sealed, able to hold a proton gradient and thus act as a hydrolytic compartment.

We suggest that exophagy is a fundamental process of monocyte-derived cells that occurs in several contexts associated with normal physiology and pathobiology. Thus, characterizing the process of extracellular degradation beyond the extensive work on bone remodeling is likely to be relevant for understanding many biological phenomena.

## MATERIALS AND METHODS

### Cells and cell culture

J774 cells and RAW 264.7 macrophage-like cells (American Type Culture Collection, Manassas, VA) were cultured and maintained in Dulbecco's Modified Eagle Medium supplemented with 10% (w/v) heat-inactivated fetal bovine serum, penicillin/streptomycin (50 units/ml) in a humidified atmosphere (5% CO_2_) at 37°C and used at low passage numbers. Cells were confirmed to be contamination-free. Bone marrow cells isolated from female C57BL/6 mice aged 6–13 weeks were differentiated for 7 days to generate BMMs by culture in the same media supplemented with 20% L-929-cell-conditioned media. Mice were housed in a pathogen-free environment at Weill Cornell Medical College and used in accordance with protocols approved by the Institutional Animal Care and Utilization Committees.

### Lipoproteins and reagents

Human LDL was prepared from donor plasma as described previously (Havel et al., 1955). LDL was labeled using succinimidyl esters of Alexa Fluor 405, Alexa Fluor 488, Alexa Fluor 546, Alexa Fluor 633 (Invitrogen, Carlsbad, CA) or CypHer 5E (GE Healthcare, Chalfont St Giles, UK). LDL was aggregated by vigorous vortexing for 30 s (Buton et al., 1999). LatA, WGA, CtB and phalloidin were purchased from Invitrogen. Filipin was purchased from Sigma-Aldrich (St Louis, MO).

### pH imaging

pH imaging was performed as described previously (Haka et al., 2009) with the following modifications. J774 cells were incubated for 1 h with agLDL which had been dual-labeled with CypHer 5E, a pH-sensitive fluorophore, and Alexa Fluor 488, a pH-insensitive fluorophore, prior to imaging. In experiments using LatA, J774 cells were incubated with dual-labeled agLDL for 30 min to establish a low pH in the compartment, and then incubated in the presence of 1 μM LatA for a further 30 min to depolymerize actin. Live cells were imaged with a Zeiss LSM 510 laser scanning confocal microscope (Thornwood, NY) using a 63× Oil 1.4 numerical aperture (NA) objective. Cell temperature was maintained at 37°C with a heated stage and objective heater. Cells were imaged in medium 2 (150 mM NaCl, 20 mM HEPES, 1 mM CaCl_2_, 5 mM KCl, 1 mM MgCl_2_) + 0.2% (w/v) glucose. Data was acquired using LSM version 4.2. *Z*-stacks were obtained using a step size of 0.46 μm (total *z* size 15.62 μm). All data were analyzed with MetaMorph image analysis software (Molecular Devices, Downingtown, PA). A binary mask was created using the Alexa Fluor 488 signal intensity and applied to both channels to remove background noise. Images were convolved with a 7×7-pixel Gaussian filter, and ratio images were produced. Ratiometric images were then used to generate a 3D reconstruction.

### Visualization of free cholesterol

RAW264.7 macrophage-like cells were incubated with Alexa546–agLDL for 1 h, labeled with Alexa555–CtB (10 μg/ml) for 5 min on ice, fixed with 3% PFA for 20 min, washed with phosphate buffered saline (PBS), incubated with filipin (50 μg/ml) for 1 h and then washed 3 times with PBS. Images were acquired on a Zeiss LSM 880, AxioObserver microscope (Thornwood, NY) equipped with a Plan-Apochromat 63× Oil 1.4 NA differential interference contrast (DIC) M27 objective. *Z*-stacks were obtained using a step size of 170 nm.

### Electron tomography

J774 cells were incubated with colloidal-gold-labeled agLDL for 1 h (Handley et al., 1981). Following incubation with agLDL the cells were fixed with a modified Karnovsky's solution containing 2.5% glutaraldehyde, 4% PFA and 0.02% picric acid, postfixed with 1% osmium tetroxide, 1.5% potassium ferricyanide, enbloc contrasted with uranyl acetate, dehydrated through a graded ethanol series and embedded in LX112 resin. En face serial sections were cut at 250 nm and picked up on formvar-coated, 4-slot copper grids. Sections were further contrasted with uranyl acetate and lead citrate. Images were acquired at the Simons Electron Microscopy Center – New York Structural Biology Center on a JEM 2100F electron microscope operating at 200 kV at a set magnification of 8000× using SerialEM tomography acquisition software (JEOL, USA Inc, Peabody, MA). The tilt range was approximately −60 to +60 degrees, using a nominal 2-degree interval with cosine adjustment. Tomogram reconstruction was performed using the Protomo software package (Winkler and Taylor, 2006).

### FIB-SEM

J774 cells were incubated with colloidal-gold-labeled agLDL for 1 h (Handley et al., 1981). After incubation with agLDL the cells were fixed with a modified Karnovsky's solution containing 2.5% glutaraldehyde, 4% PFA in 0.1M sodium cacodylate buffer (pH 7.3) containing 0.002% picric acid; postfixed with 1% osmium tetroxide, 1.5% potassium ferricyanide, enbloc contrasted with uranyl acetate, dehydrated through a graded ethanol series and embedded in LX112 resin (Ladd Research Industries, Williston, VT). The polymerized sample blocks were trimmed and thin sections were collected to identify the area of interest. The sample block was then mounted on the SEM sample holder using double-sided carbon tape (Electron Microscopy Science, Hatfield, PA) and the exposed edges of the block were electrically grounded by colloidal silver paint (Electron Microscopy Sciences). The entire surface of the specimen was then sputter coated with a thin layer of gold/palladium in a Denton Vacuum Desk V and the cells were imaged using BSE mode in a FEI Helios Nanolab 650. Images were recorded after each round of ion beam milling using the SEM beam at 3 keV and 100 pA with a working distance of 2.5 mm. Data acquisition occurred in an automated way using the Auto Slice and View G3 software (FEI, Hillsboro, OR), with an *xy* pixel size of 22.5635 nm and *z* step size of 40 nm, resulting in 990 slices with typical volumes of 43.3 µm by 19.98 µm by 39.6 µm. The segmentation and 3D reconstruction were carried out using Avizo 7.1 3D Software (FEI). For this, a median filter was applied for all image stacks and the regions of interest were manually segmented and reconstructed. The supplementary movie (Movie 3) was also created using Avizo 7.1 3D software.

### Plasma membrane labeling with CtB and WGA

J774 cells were incubated with Alexa633–agLDL for 1 h, labeled with Alexa555–CtB (10 μg/ml) for 5 min on ice, and then fixed with 3% PFA and 0.5% glutaraldehyde for 45 min and washed with PBS. Cells were then labeled with Alexa488–WGA (1 μg/ml in PBS) for 10 min at room temperature and washed with PBS. Cells were imaged using a Zeiss LSM 510 laser scanning confocal microscope using a 63×1.4 NA objective. *Z*-stacks were obtained using a step size of 0.46 μm. Images were convolved with a 5×5-pixel Gaussian filter.

### F-actin imaging

BMMs incubated with Alexa546–agLDL for 1 h were fixed with 3% PFA for 20 min and washed with PBS. Cells were next stained with Alexa488–phalloidin (0.02 U/ml) in 0.5% saponin (w/v) in PBS for 1 h, washed 3 times with PBS and then imaged. Imaging was performed on a Nikon A1R Laser Scanning Confocal Microscope (Nikon Corporation, Melville, NY) using a 60× Oil 1.4 NA objective. *Z*-stacks were obtained using a step size of 1 μm (total *z*-size of 29 μm). A 3D reconstruction from these *z*-stacks, and a movie rotating the 3D reconstruction along the *x* axis was then created using NIS-Elements C software (Nikon Corporation).

### 3D-SIM

J774 cells incubated with Alexa405–agLDL for 1 h were stained with Alexa488–CtB (10 μg/ml in ice cold media) for 15 min on an ice slurry. The cells were washed with PBS, fixed with 3% PFA for 20 min and F-actin was stained with Alexa555–phalloidin (0.02 U/ml in PBS) for 1 h. The cells were mounted in non-hardening Vectashield mounting medium (Vector Laboratories, Burlingame, CA) immediately prior to microscopy. 3D-SIM images were acquired using a DeltaVision OMX V4/Blaze system (GE Healthcare) fitted with an Olympus 100× Oil 1.40 NA UPLSAPO objective and Photometrics Evolve EMCCD cameras. The immersion oil was optimized for the 488 channel with a refractive index of 1.516 and *z*-stacks were acquired with a step size of 125 nm. SI reconstruction and image registration were performed using softWoRx v. 6.1 software (GE Healthcare).

The thickness of the sample resulted in significant scattering of the structured illumination and, thus, low signal-to-noise data. Consequently, the images were processed using ImageJ software (NIH, Bethesda, MD). First the images were smoothed using a Gaussian filter with a radius of 1 standard deviation. Next, the inherent ImageJ ‘subtract background’ command was applied with a radius of 10 pixels to enhance edges and other structures with a sudden change in intensity. Finally, the remaining noise was removed by thresholding the image such that pixels with intensities below the threshold intensity were set to zero.

### Airyscan confocal microscopy

High resolution confocal microscopy was performed on a Zeiss LSM 880, AxioObserver microscope equipped with a Plan-Apochromat 63× Oil 1.4 NA DIC M27 objective (Zeiss). *Z*-stacks were acquired using a step size of 159 nm (total *z*-size 5.44 μm). Image segmentation and surface reconstruction of the 3D Airyscan confocal microscopy images was carried out in Avizo 7.1 (FEI Visualization Sciences Group). Each image channel was segmented individually by intensity thresholding and the final surface reconstruction was generated by an overlay of the three individual surfaces.

### Actin depolymerization

J774 cells were incubated with Alexa546–agLDL for 1 h in the presence of DMSO or 1 μM LatA, labeled with Alexa488–CtB (10 μg/ml) for 3 min on ice (or left untreated to stain F-actin using Alexa488–phalloidin post-fixation), and then fixed with 3% PFA for 20 min and washed with PBS. Cells were imaged using a Zeiss LSM 510 laser scanning confocal microscope with a 40× Air 0.8 NA objective. *Z*-stacks were obtained using a step size of 0.98 μm. All data were analyzed with MetaMorph image analysis software. A binary mask was created using the Alexa546–agLDL signal intensity, and integrated Alexa488–CtB and Alexa488–phalloidin signal colocalized with the mask was quantified on a per field basis.

### LRP1 inhibition

BMMs were left untreated or pre-incubated with 1 μM RAP (ARP American Research Products, Waltham, MA) for 1 h prior to incubation for 1 h with Alexa546–agLDL in the presence of inhibitor. Cells were washed with PBS, fixed with 3% PFA for 20 min. Cells were stained for F-actin using Alexa488–phalloidin, and Alexa488–phalloidin signal colocalized with agLDL was quantified as described above.

## References

[JCS181743C1] Ameis D., Merkel M., Eckerskorn C. and Greten H. (1994). Purification, characterization and molecular cloning of human hepatic lysosomal acid lipase. *Eur. J. Biochem.* 219, 905-914. 10.1111/j.1432-1033.1994.tb18572.x 8112342

[JCS181743C2] Baron R., Neff L., Louvard D. and Courtoy P. J. (1985). Cell-mediated extracellular acidification and bone resorption: evidence for a low pH in resorbing lacunae and localization of a 100-kD lysosomal membrane protein at the osteoclast ruffled border. *J. Cell Biol.* 101, 2210-2222. 10.1083/jcb.101.6.2210 3905822 PMC2114017

[JCS181743C3] Buton X., Mamdouh Z., Ghosh R., Du H., Kuriakose G., Beatini N., Grabowski G. A., Maxfield F. R. and Tabas I. (1999). Unique cellular events occurring during the initial interaction of macrophages with matrix-retained or methylated aggregated low density lipoprotein (LDL): prolonged cell-surface contact during which ldl-cholesteryl ester hydrolysis exceeds ldl protein degradation. *J. Biol. Chem.* 274, 32112-32121. 10.1074/jbc.274.45.32112 10542246

[JCS181743C4] Caron E. and Hall A. (1998). Identification of two distinct mechanisms of phagocytosis controlled by different Rho GTPases. *Science* 282, 1717-1721. 10.1126/science.282.5394.1717 9831565

[JCS181743C5] Chellaiah M. A. (2005). Regulation of actin ring formation by rho GTPases in osteoclasts. *J. Biol. Chem.* 280, 32930-32943. 10.1074/jbc.M500154200 16006560

[JCS181743C6] Grosheva I., Haka A. S., Qin C., Pierini L. M. and Maxfield F. R. (2009). Aggregated LDL in contact with macrophages induces local increases in free cholesterol levels that regulate local actin polymerization. *Arterioscler. Thromb. Vasc. Biol.* 29, 1615-1621. 10.1161/ATVBAHA.109.191882 19556523 PMC2755184

[JCS181743C7] Haka A. S., Grosheva I., Chiang E., Buxbaum A. R., Baird B. A., Pierini L. M. and Maxfield F. R. (2009). Macrophages create an acidic extracellular hydrolytic compartment to digest aggregated lipoproteins. *Mol. Biol. Cell* 20, 4932-4940. 10.1091/mbc.E09-07-0559 19812252 PMC2785736

[JCS181743C8] Haka A. S., Singh R. K., Grosheva I., Hoffner H., Capetillo-Zarate E., Chin H. F., Anandasabapathy N. and Maxfield F. R. (2015). Monocyte-derived dendritic cells upregulate extracellular catabolism of aggregated low-density lipoprotein on maturation, leading to foam cell formation. *Arterioscler. Thromb. Vasc. Biol.* 35, 2092-2103. 10.1161/ATVBAHA.115.305843 26293468 PMC4583358

[JCS181743C9] Handley D. A., Arbeeny C. M., Witte L. D. and Chien S. (1981). Colloidal gold--low density lipoprotein conjugates as membrane receptor probes. *Proc. Natl. Acad. Sci. USA* 78, 368-371. 10.1073/pnas.78.1.368 6264440 PMC319054

[JCS181743C10] Havel R. J., Eder H. A. and Bragdon J. H. (1955). The distribution and chemical composition of ultracentrifugally separated lipoproteins in human serum. *J. Clin. Invest.* 34, 1345-1353. 10.1172/JCI103182 13252080 PMC438705

[JCS181743C11] Jurdic P., Saltel F., Chabadel A. and Destaing O. (2006). Podosome and sealing zone: specificity of the osteoclast model. *Eur. J. Cell Biol.* 85, 195-202. 10.1016/j.ejcb.2005.09.008 16546562

[JCS181743C12] Kruth H. S., Jones N. L., Huang W., Zhao B., Ishii I., Chang J., Combs C. A., Malide D. and Zhang W.-Y. (2005). Macropinocytosis is the endocytic pathway that mediates macrophage foam cell formation with native low density lipoprotein. *J. Biol. Chem.* 280, 2352-2360. 10.1074/jbc.M407167200 15533943

[JCS181743C13] Kuchibhotla S., Vanegas D., Kennedy D. J., Guy E., Nimako G., Morton R. E. and Febbraio M. (2008). Absence of CD36 protects against atherosclerosis in ApoE knock-out mice with no additional protection provided by absence of scavenger receptor A I/II. *Cardiovasc. Res.* 78, 185-196. 10.1093/cvr/cvm093 18065445 PMC2810680

[JCS181743C14] Kunjathoor V. V., Febbraio M., Podrez E. A., Moore K. J., Andersson L., Koehn S., Rhee J. S., Silverstein R., Hoff H. F. and Freeman M. W. (2002). Scavenger receptors class A-I/II and CD36 are the principal receptors responsible for the uptake of modified low density lipoprotein leading to lipid loading in macrophages. *J. Biol. Chem.* 277, 49982-49988. 10.1074/jbc.M209649200 12376530

[JCS181743C15] Kusumi A. and Suzuki K. (2005). Toward understanding the dynamics of membrane-raft-based molecular interactions. *Biochim. Biophys. Acta* 1746, 234-251. 10.1016/j.bbamcr.2005.10.001 16368465

[JCS181743C16] Labrousse A. M., Meunier E., Record J., Labernadie A., Beduer A., Vieu C., Ben Safta T. and Maridonneau-Parini I. (2011). Frustrated phagocytosis on micro-patterned immune complexes to characterize lysosome movements in live macrophages. *Front. Immunol.* 2, 51 10.3389/fimmu.2011.00051 22566841 PMC3341964

[JCS181743C17] Luxenburg C., Geblinger D., Klein E., Anderson K., Hanein D., Geiger B. and Addadi L. (2007). The architecture of the adhesive apparatus of cultured osteoclasts: from podosome formation to sealing zone assembly. *PLoS ONE* 2, e179 10.1371/journal.pone.0000179 17264882 PMC1779809

[JCS181743C18] Manning-Tobin J. J., Moore K. J., Seimon T. A., Bell S. A., Sharuk M., Alvarez-Leite J. I., de Winther M. P. J., Tabas I. and Freeman M. W. (2009). Loss of SR-A and CD36 activity reduces atherosclerotic lesion complexity without abrogating foam cell formation in hyperlipidemic mice. *Arterioscler. Thromb. Vasc. Biol.* 29, 19-26. 10.1161/ATVBAHA.108.176644 18948635 PMC2666043

[JCS181743C19] Mayor S., Rothberg K. G. and Maxfield F. R. (1994). Sequestration of GPI-anchored proteins in caveolae triggered by cross-linking. *Science* 264, 1948-1951. 10.1126/science.7516582 7516582

[JCS181743C20] McHugh K. P., Hodivala-Dilke K., Zheng M.-H., Namba N., Lam J., Novack D., Feng X., Ross F. P., Hynes R. O. and Teitelbaum S. L. (2000). Mice lacking beta3 integrins are osteosclerotic because of dysfunctional osteoclasts. *J. Clin. Invest.* 105, 433-440. 10.1172/JCI8905 10683372 PMC289172

[JCS181743C21] Moore K. J., Kunjathoor V. V., Koehn S. L., Manning J. J., Tseng A. A., Silver J. M., McKee M. and Freeman M. W. (2005). Loss of receptor-mediated lipid uptake via scavenger receptor A or CD36 pathways does not ameliorate atherosclerosis in hyperlipidemic mice. *J. Clin. Invest.* 115, 2192-2201. 10.1172/JCI24061 16075060 PMC1180534

[JCS181743C22] Nagao T., Qin C., Grosheva I., Maxfield F. R. and Pierini L. M. (2007). Elevated cholesterol levels in the plasma membranes of macrophages inhibit migration by disrupting RhoA regulation. *Arterioscler. Thromb. Vasc. Biol.* 27, 1596-1602. 10.1161/ATVBAHA.107.145086 17495238

[JCS181743C23] Nakamura I., Pilkington M. F., Lakkakorpi P. T., Lipfert L., Sims S. M., Dixon S. J., Rodan G. A. and Duong L. T. (1999). Role of alpha(v)beta(3) integrin in osteoclast migration and formation of the sealing zone. *J. Cell Sci.* 112, 3985-3993. 10547359 10.1242/jcs.112.22.3985

[JCS181743C24] Nakaya M., Kitano M., Matsuda M. and Nagata S. (2008). Spatiotemporal activation of Rac1 for engulfment of apoptotic cells. *Proc. Natl. Acad. Sci. USA* 105, 9198-9203. 10.1073/pnas.0803677105 18591655 PMC2442128

[JCS181743C25] Prashar A., Bhatia S., Gigliozzi D., Martin T., Duncan C., Guyard C. and Terebiznik M. R. (2013). Filamentous morphology of bacteria delays the timing of phagosome morphogenesis in macrophages. *J. Cell Biol.* 203, 1081-1097. 10.1083/jcb.201304095 24368810 PMC3871431

[JCS181743C26] Punturieri A., Filippov S., Allen E., Caras I., Murray R., Reddy V. and Weiss S. J. (2000). Regulation of elastinolytic cysteine proteinase activity in normal and cathepsin K-deficient human macrophages. *J. Exp. Med.* 192, 789-800. 10.1084/jem.192.6.789 10993910 PMC2193285

[JCS181743C27] Qin C., Nagao T., Grosheva I., Maxfield F. R. and Pierini L. M. (2006). Elevated plasma membrane cholesterol content alters macrophage signaling and function. *Arterioscler. Thromb. Vasc. Biol.* 26, 372-378. 10.1161/01.ATV.0000197848.67999.e1 16306428

[JCS181743C28] Sakr S. W., Eddy R. J., Barth H., Wang F. W., Greenberg S., Maxfield F. R. and Tabas I. (2001). The uptake and degradation of matrix-bound lipoproteins by macrophages require an intact actin cytoskeleton, Rho family GTPases, and myosin ATPase activity. *J. Biol. Chem.* 276, 37649-37658. 10.1074/jbc.M105129200 11477084

[JCS181743C29] Singh A. V., Batuwangala M., Mundra R., Mehta K., Patke S., Falletta E., Patil R. and Gade W. N. (2014). Biomineralized anisotropic gold microplate–macrophage interactions reveal frustrated phagocytosis-like phenomenon: a novel paclitaxel drug delivery vehicle. *ACS Appl. Mater. Interfaces* 6, 14679-14689. 10.1021/am504051b 25046687

[JCS181743C30] Stenbeck G. (2002). Formation and function of the ruffled border in osteoclasts. *Semin. Cell Dev. Biol.* 13, 285-292. 10.1016/S1084952102000587 12243728

[JCS181743C31] Swanson J. A. and Hoppe A. D. (2004). The coordination of signaling during Fc receptor-mediated phagocytosis. *J. Leukoc. Biol.* 76, 1093-1103. 10.1189/jlb.0804439 15466916

[JCS181743C32] Tzircotis G., Braga V. M. M. and Caron E. (2011). RhoG is required for both FcgammaR- and CR3-mediated phagocytosis. *J. Cell Sci.* 124, 2897-2902. 10.1242/jcs.084269 21878497

[JCS181743C33] Winkler H. and Taylor K. A. (2006). Accurate marker-free alignment with simultaneous geometry determination and reconstruction of tilt series in electron tomography. *Ultramicroscopy* 106, 240-254. 10.1016/j.ultramic.2005.07.007 16137829

[JCS181743C34] Zhang W.-Y., Gaynor P. M. and Kruth H. S. (1997). Aggregated low density lipoprotein induces and enters surface-connected compartments of human monocyte-macrophages: uptake occurs independently of the low density lipoprotein receptor. *J. Biol. Chem.* 272, 31700-31706. 10.1074/jbc.272.50.31700 9395512

